# Awareness and Attitude of Surgeons regarding Dental Erosion on Patients Who Underwent Bariatric Surgery

**DOI:** 10.1155/2022/1812715

**Published:** 2022-02-21

**Authors:** Omir Aldowah

**Affiliations:** Prosthetic Dental Science Department, Faculty of Dentistry, Najran University, Najran 66446, Saudi Arabia

## Abstract

**Background:**

The obesity epidemic is considered one of the main challenges for modern medicine. It has been proven that bariatric surgery is more effective than nonsurgical interventions to manage weight-related comorbidities. The general surgeon needs to understand how tooth erosion advances in gastroesophageal reflux in people who have had bariatric surgery. Dental erosion caused due to gastric reflux begins with the enamel, which is the tough, protective coating that covers our teeth. While it is tough, it is prone to an extremely acidic environment with a low pH, where it begins to soften and demineralize, gradually wearing away and exposing the more sensitive areas of the tooth. Because of the growing popularity of this subspecialty, general surgeons should develop a basic clinical and surgical understanding of these standard procedures and complications, regardless of their interest in obesity surgery.

**Aim:**

This cross-sectional study aimed to assess the awareness and attitude of surgeons regarding dental erosion on patients who underwent bariatric surgery.

**Materials and Methods:**

This cross-sectional study was conducted on general surgeons from different regions of Saudi Arabia who perform bariatric surgeries. Data collection was done by sending the questionnaire to the general surgeons by different means of social media (WhatsApp, e-mail, Facebook, etc.), and it was also distributed through the Saudi Arabia Society of Metabolic and Bariatric Surgery.

**Results:**

A total of 25 general surgeons responded to the survey. Half of the respondents know what dental erosion is, 52%. Most of them, 72%, are not aware of the relationship between dental erosion and acid reflux or vomiting. They reported that 52% of patients complain of gastroesophageal reflux disorder.

**Conclusion:**

The general surgeons had inadequate awareness and attitude regarding dental erosion on bariatric surgery patients. Our findings suggest that a lack of adequate awareness and a negative attitude among general surgeons are grounds for concern and that more should be done to avoid oral health complications.

## 1. Introduction 

The obesity epidemic is considered one of the main challenges for modern medicine. World Health Organization data showed that more than 10% of the adult population is obese worldwide. Several studies confirmed that bariatric surgery is the gold standard for the treatment of obesity. Also, it has been proven that bariatric surgery is more effective than nonsurgical interventions to manage weight-related comorbidities [1, 2]. In the fourth report of the largest registry on bariatric surgery worldwide, 394,431 bariatric surgeries were done in 51 countries [3]. Surgery for extreme obesity has been shown to improve the control and treatment of comorbidities like hypertension, coronary heart disease, diabetes, sleep apnea, osteoarthritis, and dyslipidemia.

Additionally, it improves the anxiety and self-esteem of bariatric surgery patients significantly [4]. Obesity and oral health are becoming increasingly popular topics of discussion. According to scientific research, obesity has been linked to oral disorders such as tooth xerostomia, erosion, oral infectious conditions (tooth decay and periodontitis), and dentin hypersensitivity [5]. Typically, bariatric patients have difficulty swallowing fluids due to the surgery's stomach alterations, resulting in reduced salivary flow. Furthermore, saliva's buffering role is harmed, increasing the likelihood of tooth decay and tooth erosion in bariatric patients [6, 7]. However, when simple carbohydrates are consumed, gastroplasty can cause side effects such as vomiting, epigastric pain, nausea, hypoglycemic, and flushing symptoms, sometimes known as the “dumping” syndrome.

Chronic vomiting, gastroesophageal reflux disease, and regurgitation are pervasive side effects of gastric restrictive bariatric surgery [8]. Several studies reported that gastroesophageal reflux diseases could be increased after bariatric surgery [9, 10]. Moreover, postoperative nausea and vomiting are common after laparoscopic bariatric surgery [11]. Marsicano et al. [12] stated that prolonged vomiting was reported by 56.3% at least once weekly. Similarly, long-term vomiting at least once weekly in a year time was reported by Bianciardi [13]. Even though the mouth cavity is physically and physiologically a part of the digestive system, the possible harmful consequences of gastric surgery on human dentition are frequently overlooked [14]. Dental erosion, aphthous ulcers, burning mouth feeling, tooth hypersensitivity, low salivary flow, and a sour taste in the mouth are more common in gastroesophageal reflux disease or chronic acid reflux patients [15]. These factors are linked to the action of an acidic pH and dietary alterations, which are typically observed with the consumption of liquid-pasty foods, resulting in the breakdown of mineralized dental tissues [16]. According to the Basic Erosive Examination scoring system, the dental erosion is classified based on the score and criteria for wear—score 0: no loss of surface, score 1: initial loss of surface texture (slight wear), score 2: distinct defect, hard tissue loss <50% of the surface texture (dentin is frequently involved), and score 3: hard tissue loss >50% of the surface area (moderate to severe lesion) [17, 18].

Because of the growing popularity of this subspecialty, general surgeons should develop a basic clinical and surgical understanding of these standard procedures and complications, regardless of their interest in obesity surgery. They may be called upon to deal with acute or chronic complications of bariatric surgery during their practice [19]. Because of the findings, it is essential to understand how tooth erosion advances in gastroesophageal reflux in people who have had bariatric surgery because the literature on the subject is still inconclusive. An inquiry was deemed necessary because bariatric surgery can cause repeated gastroesophageal reflux and vomiting, which promotes teeth contact with gastric acids. We hypothesized that general surgeons have a positive attitude and are aware of the fact that dental erosion is more prevalent in the bariatric patients because of the exposure to gastric reflux. This cross-sectional study aimed to assess the awareness and attitude of surgeons regarding dental erosion on patients who underwent bariatric surgery.

## 2. Methodology

This cross-sectional study was conducted on general surgeons who perform bariatric surgeries. This survey includes 25 general surgeons from different regions of Saudi Arabia. The inclusion criteria include general surgeons working in different sectors like the government sector and private sector. The exclusion criteria were general surgeons working in academics, pediatric surgeons, vascular surgeons, and general surgeons who do not want to participate in the survey. This study was approved by the Scientific Research Ethical Committee of Najran University, Saudi Arabia, with reference number 442-40-34293-DS. The study duration was from December 2020 to May 2021.

Data collection was done by sending a questionnaire to the general surgeons by different means of social media (WhatsApp, e-mail, Facebook, etc.), and it was also distributed through the Saudi Arabia Society of Metabolic and Bariatric Surgery. No sample size calculation was carried out because of the maximum sample size that could be recruited. A convenient sampling technique was used to perform the survey. Based on the knowledge of bariatric surgeons at a big tertiary center, an initial questionnaire was prepared. Following that, a focus group with five bariatric surgeons from a tertiary academic center was held. The focus group feedback was used to improve and validate the questionnaire. The final questionnaire included 15 questions designed to assess bariatric surgeons' knowledge and attitudes toward dental erosion. The questionnaire contains queries like working in the government or private sector, the rank of the surgeons, years of experience, and the questions that assess the awareness and attitude of surgeons regarding dental erosion on patients who underwent bariatric surgery.

### 2.1. Statistical Analysis

A statistical software program (SPSS v.22) was used for all statistical analyses, and descriptive statistics were reported for continuous variables. The experience of the doctors was categorized into 2 groups: less or equal to 10 years (G1) and more significant and equal to 11 years (G2). Then, the association of categorized experience with other responses was determined. Since the sample size was not large enough, the chi-square test was not valid in many attempts to find the association.

## 3. Results

The questionnaire was sent to 30 general surgeons by different means of social media; a total of 25 general surgeons responded (response rate 83.33%) and were included in the study. The 3 questionnaires were returned because of incorrect e-mail addresses, and 2 questionnaires were returned due to incomplete data and were excluded from the study. The mean years of experience is 11.0 ± 7.5 [R: 2–25] years. The mean number of surgeries per month is 24.0 ± 19.6 [R: 2–80] surgeries. 12% of surgeons performed bariatric surgeries in the private sector. Other surgeons performed them on governmental 48% or both sectors 40%. 88% of respondents are consultants. Half of the respondents know what dental erosion is, 52%. Most of them, 72%, are not aware of the relationship between dental erosion and acid reflux or vomiting. They reported that 52% of patients complain of gastroesophageal reflux disorder. However, patients who underwent bariatric surgeries rarely complained of prolonged vomiting, 76%. Also, 76% of surgeons are not aware that tooth hypersensitivity could indicate dental erosion. Similarly, 60% of the surgeons know whether bariatric surgery could induce dental erosion. Only 44% of the surgeons agreed that dental health could affect patient satisfaction after bariatric surgery.

Their opinions regarding that dentists should have a role with patient management before and after bariatric surgery tend to agree (strongly agree = 5, agree = 8). Most of the surgeons prescribed antacids/proton pump inhibitors for 8 weeks, 76%. Few surgeons recommend the patients to visit the dentist after surgery, 20%. Many surgeons (84%) neglect recommending the patients to follow or apply dental erosion prevention measures. Descriptive statistics of response are presented in Table 1.

Fifty percent of the respondents indicated that their experience was less or equal to 10 years. Sixty-one percent of the respondents of experience group G1 mentioned that dental erosion is a loss of tooth structure. This percentage was higher than the respondents of group G2 but not statistically significant (*p*=0.319). Seventy-two percent of the doctors did not know that dental erosion that caused by acid reflux or vomiting. Even though group G2 showed a significantly higher percentage than group G1 for the option “I do not know” (*p*=0.02), chi-square is not valid due to low sample size. There was no significant difference between group G1 and group G2 for patients who complain of prolonged vomiting. Similarly, no significant difference was observed between group G1 and group G2 for the question “Tooth hypersensitivity is an indicator.” None of the other responses show any significant difference between group G1 and group G2 (Table 2).

Respondents considered all dental erosion preventive measures. However, they were more concerned about the medical management of GERD 84%, followed by using fluoride in a gel, toothpaste, or varnish 24%, enhancing salivary flow as needed 20%, and covering teeth with an occlusal splint made in hard acrylic 8% (Table 3).

## 4. Discussion

Bariatric surgery is the most successful treatment for severe obesity for millions of bariatric patients worldwide, resulting in significant weight loss [17]. Bariatric surgery treatment and long-term loss of weight improved patients' life quality and decreased various comorbidities linked with surgery, such as diabetes, dyslipidemia, hypertension, osteoarthritis, cardiovascular disease, and sleep apnea [16]. However, several adverse effects might impact some aspects of oral health, such as tooth erosion [20]. In the medical literature, general surgeons' oral complications, awareness, and attitude regarding dental erosions and side effects of bariatric surgery have not been well reported. To the best of our knowledge, no study has been done in Saudi Arabia among general surgeons. This cross-sectional study aimed to assess the awareness and attitude of surgeons regarding dental erosion on patients who underwent bariatric surgery.

Our study results revealed that the mean years of working experience of general surgeons was 11.0 ± 7.5 [R: 2–25] years and the mean number of surgeries per month was 24.0 ± 19.6 [R: 2–80] surgeries. 12% of surgeons performed bariatric surgeries in the private sector. Other surgeons performed them on governmental 48% or both sectors 40%. 88% of respondents were consultants. These results show that the general surgeons were having good experience and exposure to bariatric surgeries. The number of bariatric procedures conducted in Saudi Arabia has increased significantly, forcing healthcare facilities, both private and public, to strengthen their supplies, resources, and expertise in this field within various healthcare settings. The bariatric surgeons had access to adequate tools and equipment for managing obese patients, and they knew whom to call if they had any problems managing patients with morbid obesity. This is in agreement with the study conducted among the Saudi population [21].

Further, this study shows that around half of the respondents, 48%, do not know what dental erosion is, and 72% do not know that acid reflux causes dental erosion. This is evident from a study conducted among the Indian population, where 52% of participants did not know what dental erosion is, and 66% did not know that acid reflux causes dental erosion [22]. The erosion of teeth occurs when the pH of the surface falls below 5.5 from the acid reflux due to GERD, regurgitation, and chronic vomiting, resulting in the loss of the outermost surface of the enamel. According to De Moura Rec [6], 90% of bariatric surgery patients reported involuntary or voluntary regurgitation episodes capable of causing erosive tooth loss [6]. So, bariatric surgeons should have a piece of adequate knowledge about the dental erosions that are caused due to the complications of bariatric surgery. Dental erosion can be prevented among bariatric patients by reducing the post-bariatric surgery complications through therapeutic management and awareness among patients about the dietary habits after surgery to avoid using acidic foods and soft drinks with low pH levels [23, 24]. As a result, the preoperative and postoperative follow-up guidelines of patients undergoing bariatric surgery are critical due to the multifactorial nature of the procedure.

Furthermore, 52% of general surgeons rarely know that patients complain about gastrointestinal reflux, 76% and 72% of surgeons rarely knew that patients complain of chronic vomiting and teeth hypersensitivity, respectively. Marsicano did a study in 2011 that reported that, after 3 months of bariatric surgery, the percentage of tooth erosion in enamel and dentin was 12.5% and 87.5% and, after 6 months of the bariatric surgery, the percentage of patients showed 100% of tooth erosion involving the dentin. According to Heling et al. [14], 37% of the patients had increased dentin sensitivity after bariatric surgery. This information, which could be linked to the possibility of dentin lesions, was mentioned based on the patient's perception. Therefore, the bariatric surgeons must consider their participation in bariatric CME, such as attending conferences and reviewing the peer journal articles to enhance their knowledge and management of postsurgical complications [25].

Our study results revealed that 60% of general surgeons do not know whether bariatric surgery could induce dental erosion. Only 44% of the surgeons agreed that dental health could affect patient satisfaction after bariatric surgery. This agrees with the findings in a study conducted among surgeons of Canada, where 41.6% of surgeons were confident of managing complications of bariatric surgery [26]. Our findings indicate a greater need for improved medical education for trainees and more general surgeon education in this area. As a result, formalized education programs could help general surgeons improve obesity treatment and postoperative care, ultimately improving patient outcomes. It appears that many general surgeons in Saudi Arabia would benefit from expanded CME resources in bariatric surgical care.

Further, our study findings show that few general surgeons recommend that patients visit the dentist after surgery, 20%. Most of the surgeons prescribed antacids/proton pump inhibitors for 8 weeks, 76%. Many surgeons (84%) neglect recommending the patients to follow or apply dental erosion prevention measures. Their opinions regarding that dentists should have a role with patient management before and after bariatric surgery tend to agree (strongly agree = 5, agree = 8). Even though bariatric surgery might produce systemic and oral alterations, the health advantages to patients outweigh the risks before surgery and during postoperative follow-up, including a dentist on the medical team to examine the oral status and execute preventative measures, treatment, and control of oral symptoms [16].

Furthermore, the study findings revealed that 50% of general surgeons had less or equal to 10 years of experience, most of them (88%) had consultant rank, and 48% of surgeries were operated in the government sector. The key elements to consider are the general surgeon's skill and experience. A large body of evidence suggests that some surgeons do better than others, with some studies revealing significant differences in risk-adjusted patient death rates between general surgeons. The operating surgeon's technical expertise may be a significant factor in many surgeries' outcomes [27]. After bariatric surgery, general surgeons and dental teams must evaluate potential dental problems and provide their patients with the necessary information and instructions for oral cleanliness, healthy eating habits, and frequent dental health monitoring by a dentist or dental hygienist [28]. The formation of such complete bariatric teams could be crucial to the success of bariatric surgeries and decrease postsurgical complications. This strategy may be beneficial in motivating these people to undertake behavioral changes that will improve their chances of success [29]. Among the general surgeons, only 52% knew that dental erosion is the loss of tooth structure, and 72% did not know that tooth erosion is caused by acid reflux or vomiting. The general surgeons reported that 52% of patients complained about gastrointestinal reflux disorder, and 72% of surgeons do not know about tooth hypersensitivity. A study done by Heling et al. [14] shows that 74% of patients suffering from dental erosion complained about gastrointestinal reflux or chronic vomiting and were significantly associated with tooth hypersensitivity.

However, results from the present study show that 72% of general surgeons never asked their patients about the symptoms of dental disease, 64% of general surgeons never asked them to visit the dentist for follow-up after the surgery, and 60% of them did not follow the dental erosion prevention. Most patients with postsurgical bariatric complications should be referred to a bariatric surgery center of excellence, whose surgeons and facilities are equipped to treat and care for bariatric surgery patients. However, not all bariatric surgery centers of excellence are easily accessible, and general surgeons frequently treat patients [30]. To acquire a more objective finding, patient-reported outcomes should be recorded before and after bariatric surgery to rule out the possibility that people who have this type of surgery are no different from people who do not, in terms of oral health and oral health-related quality of life. Even better, clinical evaluations may support such research to determine their dental health status [31].

This study shows that 76% of general surgeons prescribed antacids/proton pump inhibitors for 8 weeks after the bariatric surgery, and almost all the surgeons advised their patients to visit a nutritionist after the surgery. Every patient should be examined before and after surgery for their ability to adhere to nutrition and lifestyle changes, according to the American Association of Clinical Endocrinologists' standards. Appropriate supplementation in combination with dietary education can be effective in preventing malnutrition [32]. Patients should be trained on the principles of moderate meal progression by an experienced dietitian before bariatric surgeries, depending on the bariatric strategy used to decrease postsurgical complications. Multidisciplinary supervision necessitates effective collaboration among general surgeons, internists, psychiatrists, and dietitians, but the patient is the most critical player in the fight against malnutrition, weight gain, and other bariatric surgery problems [32].

The majority of the general surgeons, 84%, reported that medical management of GERD or chronic vomiting is the preventive measure that can be taken to avoid dental erosions. The proton pump inhibitor or H2 blockers, commonly taken to treat GERD, are also used to treat or avoid problems (anastomotic ulcer) and gastrointestinal symptoms that may arise following bariatric surgery [33]. This leads to a decrease in acid reflux into the mouth that causes dental erosions. Also, 24% of general surgeons reported that fluoride in a gel, toothpaste, or varnish could prevent dental erosions. Appropriate education on dental health must instill in patients the importance of maintaining their dental health following surgery. This should involve proper mouth hygiene and dietary habits and the use of fluoride and dental appointments. After surgery, patients should see a dentist or a dental hygienist as soon as their teeth become hypersensitive [14].

There are a few limitations to this study. The replies may not represent all general surgeons' perspectives. Selection bias may have influenced the results of our study, as respondents who were more familiar with the topic matter were more likely to reply to the survey. The responses of general surgeons may have been influenced by social desirability bias, which led them to give what they thought was the “right” answer about their practices. Although we asked the general surgeons about their practices, we did not ask other bariatric surgery healthcare teams (such as physician assistants or nurse practitioners).

### 4.1. Limitations

The primary limitation of our study was whether this sample size of general surgeons is representative of all Saudi Arabia performing the bariatric surgeries. Second was respondent's fatigue, whether the general surgeons had appropriate time to answer the questions given to them because of their busy schedule. Only few studies are available in literature on this topic, which limits to identify the research gaps and to present the need for further development in the area.

## 5. Conclusion

We conclude that, in this survey, the general surgeons had inadequate awareness and attitude regarding dental erosion on patients who underwent bariatric surgery. General surgeons should understand the implications given in this study. Our findings suggest that a lack of adequate awareness and a negative attitude among general surgeons are grounds for concern and that more should be done to avoid oral health complications. We recommend that multidisciplinary surveillance necessitates effective collaboration among general surgeons, dentists, internists, psychiatrists, and dietitians to avoid post-bariatric surgery problems.

## Figures and Tables

**Table 1:** Descriptive statistics of responses from general surgeons.

Items	Number	Percentage
Where do you do the bariatric surgery?		
Both	10	40.0
Governmental sector	12	48.0
Private sector	3	12.0

Ranking		
Consultant	22	88.0
Resident under training	1	4.0
Senior registrar	2	8.0

Dental erosion is loss of tooth structure		
I do not know	12	48.0
Yes	13	52.0

Dental erosion that caused by acid reflux or vomiting		
I do not know	18	72.0
No	2	8.0
Yes	5	20.0

Patients complain of gastroesophageal reflux disorder		
Often	13	52.0
Rarely	12	48.0

Patients complain of prolonged vomiting		
Often	6	24.0
Rarely	19	76.0

Tooth hypersensitivity is an indicator		
I do not know	18	72.0
No	1	4.0
Yes	6	24.0

The dental erosion can be increased		
I do not know	15	60.0
No	3	12.0
Yes	7	28.0

Patient satisfaction after bariatric surgery		
Agree	11	44.0
Disagree	4	16.0
Neutral	2	8.0
Strongly agree	6	24.0
Strongly disagree	2	8.0

Patient management before & after bariatric surgery		
Agree	8	32.0
Disagree	3	12.0
Neutral	5	20.0
Strongly agree	5	20.0
Strongly disagree	4	16.0

Antacids/proton pump inhibitors for bariatric surgery		
4 weeks	3	12.0
6 weeks	3	12.0
8 weeks	19	76.0

Ask symptoms of dental diseases		
No	18	72.0
No need to ask	2	8.0
Yes	5	20.0
Visit their dentists for follow-up after the surgery		

No	16	64.0
Not required	4	16.0
Yes	5	20.0

Visit nutritionist after the surgery		
Yes	25	100.0

Follow dental erosion preventions		
No	15	60.0
Not required	4	16.0
Yes	6	24.0

**Table 2:** Responses categorized by the years of experience of general surgeons.

Items	Experience		Total	*P* value
	≥11	≤10		
Where do you do the bariatric surgery?				
Both	5 (38.5)	5 (41.7)	10 (40.0)	0.116
Governmental sector	8 (61.5)	4 (33.3)	12 (48.0)	
Private sector	0 (0.0)	3 (25.0)	3 (12.0)	

Ranking				
Consultant	10 (76.9)	12 (100.0)	22 (88.0)	0.116
Resident under training	1 (7.7)	0 (0.0)	1 (4.0)	
Senior registrar	2 (15.4)	0 (0.0)	2 (8.0)	

Dental erosion is loss of tooth structure				
I do not know	5 (38.5)	7 (58.3)	12 (48.0)	0.319^*∗*^
Yes	8 (61.5)	5 (41.7)	13 (52.0)	

Dental erosion that caused by acid reflux or vomiting				
I do not know	7 (53.8)	11 (91.7)	18 (72.0)	0.020
No	1 (7.7)	1 (8.3)	2 (8.0)	
Yes	5 (38.5)	0 (0.0)	5 (20.0)	

Patients complain of gastroesophageal reflux disorder				
Often	8 (61.5)	5 (41.7)	13 (52.0)	0.319^*∗*^
Rarely	5 (38.5)	7 (58.3)	12 (48.0)	

Patients complain of prolonged vomiting				
Often	3 (23.1)	3 (25.0)	6 (24.0)	0.910
Rarely	10 (76.9)	9 (75.0)	19 (76.0)	

Tooth hypersensitivity is an indicator				
I do not know	9 (69.2)	9 (75.0)	18 (72.0)	0.363
No	0 (0.0)	1 (8.3)	1 (4.0)	
Yes	4 (30.8)	2 (16.7)	6 (24.0)	

The dental erosion can be increased				
I do not know	9 (69.2)	6 (50.0)	15 (60.0)	0.088
No	0 (0.0)	3 (25.0)	3 (12.0)	
Yes	4 (30.8)	3 (25.0)	7 (28.0)	

Patient satisfaction after bariatric surgery				
Agree	7 (53.8)	4 (33.3)	11 (44.0)	0.259
Disagree	1 (7.7)	3 (25.0)	4 (16.0)	
Neutral	1 (7.7)	1 (8.3)	2 (8.0)	
Strongly agree	4 (30.8)	2 (16.7)	6 (24.0)	
Strongly disagree	0 (0.0)	2 (16.7)	2 (8.0)	

Patient management before & after bariatric surgery				
Agree	6 (46.2)	2 (16.7)	8 (32.0)	0.428
Disagree	1 (7.7)	2 (16.7)	3 (12.0)	
Neutral	2 (15.4)	3 (25.0)	5 (20.0)	
Strongly agree	3 (23.1)	2 (16.7)	5 (20.0)	
Strongly disagree	1 (7.7)	3 (25.0)	4 (16.0)	

Antacids/proton pump inhibitors for bariatric surgery				
4 weeks	1 (7.7)	2 (16.7)	3 (12.0)	0.707
6 weeks	2 (15.4)	1 (8.3)	3 (12.0)	
8 weeks	10 (76.9)	9 (75.0)	19 (76.0)	

Ask symptoms of dental diseases				
No	9 (69.2)	9 (75.0)	18 (72.0)	0.231
No need to ask	2 (15.4)	0 (0.0)	2 (8.0)	
Yes	2 (15.4)	3 (15.0)	5 (20.0)	

Visit their dentists for follow-up after the surgery				
No	8 (61.5)	8 (66.7)	16 (64.0)	0.547
Not required	3 (23.1)	1 (8.3)	4 (16.0)	
Yes	2 (15.4)	3 (25.0)	5 (20.0)	

Visit nutritionist after the surgery				
Yes	13 (100.0)	12 (100.0)	25 (100.0)	25

Follow dental erosion preventions				
No	9 (69.2)	6 (50.0)	15 (60.0)	0.104
Not required	3 (23.1)	1 (8.3)	4 (16.0)	
Yes	1 (7.7)	5 (41.7)	6 (24.0)	

**Table 3:** Descriptive statistics of preventive measures.

Preventive measures	*N*	Percentage
Covering teeth with an occlusal splint made in hard acrylic	2	8
Medical management for GERD	21	84
Enhance salivary flow as needed	5	20
Using fluoride in form of gel, toothpaste, or varnish	6	24
Delay tooth brushing by enough time after acid attack either from vomiting or acid regurgitation	2	8
Other	2	8

## Data Availability

The data presented in this study are available upon request to the author.
